# Supplementary material for: Polyphenol Composition and Antioxidant Potential of Instant Gruels Enriched with Lycium barbarum L. Fruit
Source: Molecules. 2020 Oct 3;25(19):4538. doi: 10.3390/molecules25194538 (PMC7583975; doi:10.3390/molecules25194538)

Supplementary material

Polyphenol Composition and Antioxidant Potential of Instant Gruels Enriched with *Lycium barbarum* L. Fruit

Marta Olech [1,*], Kamila Kasprzak [2,*], Agnieszka Wójtowicz [3,*], Tomasz Oniszczuk [3,*], Renata Nowak [1], Monika Waksmundzka-Hajnos [2], Maciej Combrzyński [3], Marek Gancarz [4], Iwona Kowalska [5], Anna Krajewska [6] and Anna Oniszczuk [2,*]

1 Department of Pharmaceutical Botany, Medical University of Lublin, 20-093 Lublin, Poland; renata.nowak@umlub.pl

2 Department of Inorganic Chemistry, Medical University of Lublin, 20-093 Lublin, Poland; monika.waksmundzka-hajnos@umlub.pl

3 Department of Thermal Technology and Food Process Engineering, University of Life Sciences in Lublin, 20-612 Lublin, Poland; maciej.combrzynski@up.lublin.pl

4 Institute of Agrophysics, Polish Academy of Sciences, 20-290 Lublin, Poland; m.gancarz@ipan.lublin.pl

5 Department of Biochemistry and Crop Quality, Institute of Soil Science and Plant Cultivation, State Research Institute, 24-100 Puławy, Poland; ikowalska@iung.pulawy.pl

6 Department of Integrated Paediatric Dentistry, Medical University of Lublin, 20-094 Lublin, Poland; anna.krajewska1@umlub.pl

* Correspondence: marta.olech@umlub.pl (M.O.); kasprzak.kamila.k@o2.pl (K.K.); agnieszka.wojtowicz@up.lublin.pl (A.W.); tomasz.oniszczuk@up.lublin.pl (T.O.); anoniszczuk@o2.pl (A.O.)

Abstract: Goji fruit (*Lycium barbarum* L.) has been identified as a polyphenolic compound plant source of noted richness. It also contains polysaccharides, carotenoids, vitamins and minerals, fatty and organic acids. The purpose of the presented research was to produce innovative instant corn gruels with various dry goji berry contents (1, 3 and 5%), to determine the level of included polyphenolic compounds (including individual free phenolic acids) and to assess the antioxidant properties of these functional-food products. A further objective was to identify the optimum value of one of the most important production parameter, the rotational speed of the extruder's screw during gruel processing. The undertaken chromatographic analysis (LC-ESI-MS/MS) showed a wide variety of available phenolic acids. In the samples with 5% addition of fruit, eight phenolic acids were detected, whereas in the corn gruel without additives, only five were noted. The antioxidant activity, the content of free phenolic acids and the sum of polyphenols increased with increase of the functional additive. For all goji content, screw speeds of 100 and 120 rpm rather than 80 rpm resulted in higher polyphenol amounts and greater Trolox equivalent antioxidant capacity, as well as higher ability to scavenge DPPH.

Keywords: dietary polyphenols; liquid chromatography; functional food, instant gruels; goji fruit; antioxidant activity; extrusion-cooking; processing parameters.

Figure S1. Extracted LC-MS-MRM chromatogram of phenolic acids found in instant gruels with addition of 5% goji fruits; screw speed 100 rpm. MRM transitions are given in brackets: 1 - protocatechuic (m/z 152.9 > 107.8); 2 - *trans*-caffeic (m/z 178.7 > 134.9); 3 - 4-hydroxy-benzoic (m/z 136.9 > 93); 4 - salicylic (m/z 136.9 > 93); 5 - gentisic (m/z 152.8 > 107.9); 6 - *p*-coumaric (m/z 162.8 > 119); 7 - ferulic (m/z 192.8 > 133.9); 8 - isoferulic acid (m/z m/z 192.8 > 133.9).

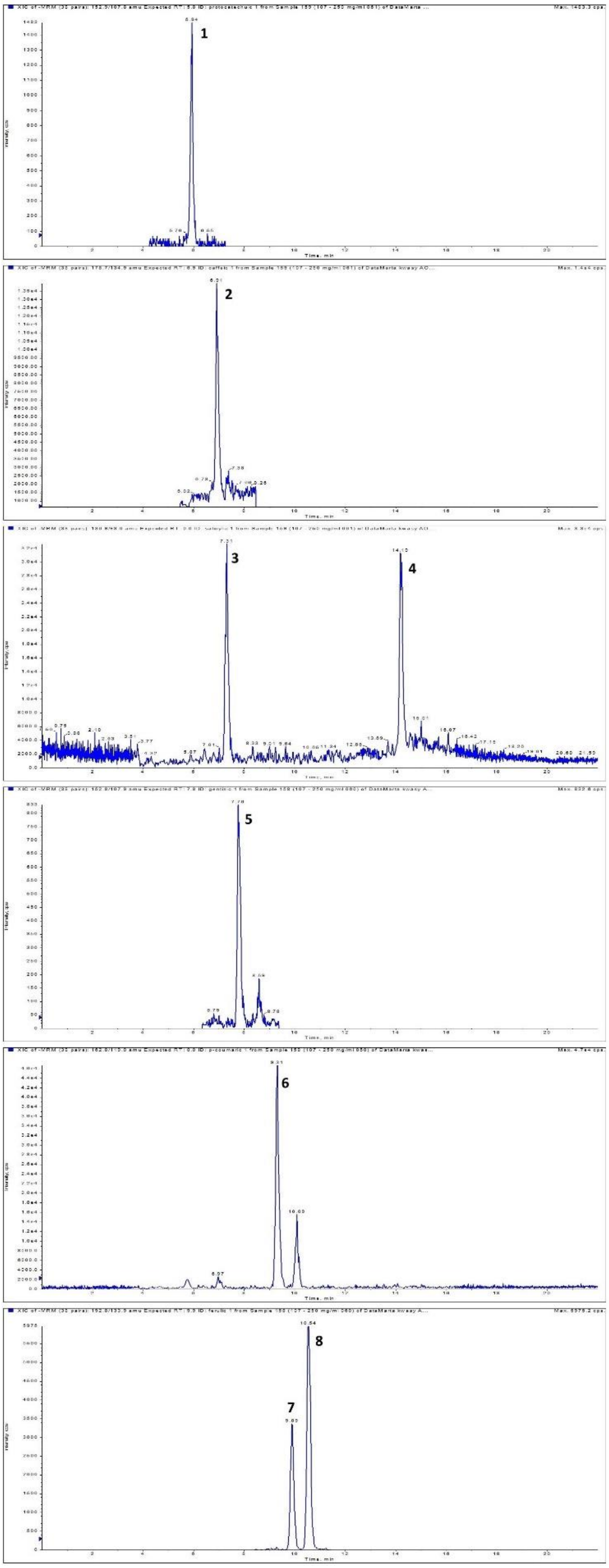

Supplement: Supplementary file 1 [file molecules-25-04538-s001.pdf]
